# PANDEMIC POLICIES MOVING FORWARD: WHAT HAVE WE LEARNED

**DOI:** 10.1093/geroni/igac059.1140

**Published:** 2022-12-20

**Authors:** Brian Lindberg

## Abstract

Leading aging and health policy advocates will present their findings and viewpoints regarding pandemic and post-pandemic policy and programmatic changes and innovations. Issues will include elder justice, home and community-based services, Medicaid, nursing home care, and social isolation. The group will discuss what has changed and how will programs and services be different in the future.

